# Predicting COPD Readmission: An Intelligent Clinical Decision Support System

**DOI:** 10.3390/diagnostics15030318

**Published:** 2025-01-29

**Authors:** Julia López-Canay, Manuel Casal-Guisande, Alberto Pinheira, Rafael Golpe, Alberto Comesaña-Campos, Alberto Fernández-García, Cristina Represas-Represas, Alberto Fernández-Villar

**Affiliations:** 1 Fundación Pública Galega de Investigación Biomédica Galicia Sur, Hospital Álvaro Cunqueiro, 36312 Vigo, Spain; julia.lopez@iisgaliciasur.es; 2 NeumoVigo I+i Research Group, Galicia Sur Health Research Institute (IIS Galicia Sur), SERGAS-UVIGO, 36312 Vigo, Spain; cristina.represas.represas@sergas.es (C.R.-R.); jose.alberto.fernandez.villar@sergas.es (A.F.-V.); 3 Centro de Investigación Biomédica en Red, CIBERES ISCIII, 28029 Madrid, Spain; 4 Department of Design in Engineering, University of Vigo, 36208 Vigo, Spain; alberto.gomes.pinheira@uvigo.es (A.P.); acomesana@uvigo.es (A.C.-C.); 5 Design, Expert Systems and Artificial Intelligent Solutions Group (DESAINS), Galicia Sur Health Research Institute (IIS Galicia Sur), SERGAS-UVIGO, 36312 Vigo, Spain; 6 Department of Computer Engineering, Superior Institute of Engineering of Porto, 4249-015 Porto, Portugal; 7 Pulmonary Department, Hospital Lucus Augusti, 27003 Lugo, Spain; rafael.golpe.gomez@sergas.es; 8 Servicio de Diagnóstico por Imagen, Hospital Ribera Povisa, 36211 Vigo, Spain; alberto.fernandez.garcia@outlook.es; 9 Pulmonary Department, Hospital Álvaro Cunqueiro, 36312 Vigo, Spain

**Keywords:** COPD, machine learning, expert systems, fuzzy logic, intelligent systems, artificial intelligence, clinical decision-making, Wang–Mendel algorithm

## Abstract

**Background:** COPD is a chronic disease characterized by frequent exacerbations that require hospitalization, significantly increasing the care burden. In recent years, the use of artificial intelligence-based tools to improve the management of patients with COPD has progressed, but the prediction of readmission has been less explored. In fact, in the state of the art, no models specifically designed to make medium-term readmission predictions (2–3 months after admission) have been found. This work presents a new intelligent clinical decision support system to predict the risk of hospital readmission in 90 days in patients with COPD after an episode of acute exacerbation. **Methods:** The system is structured in two levels: the first one consists of three machine learning algorithms —Random Forest, Naïve Bayes, and Multilayer Perceptron—that operate concurrently to predict the risk of readmission; the second level, an expert system based on a fuzzy inference engine that combines the generated risks, determining the final prediction. The employed database includes more than five hundred patients with demographic, clinical, and social variables. Prior to building the model, the initial dataset was divided into training and test subsets. In order to reduce the high dimensionality of the problem, filter-based feature selection techniques were employed, followed by recursive feature selection supported by the use of the Random Forest algorithm, guaranteeing the usability of the system and its potential integration into the clinical environment. After training the models in the first level, the knowledge base of the expert system was determined on the training data subset using the Wang–Mendel automatic rule generation algorithm. **Results:** Preliminary results obtained on the test set are promising, with an AUC of approximately 0.8. At the selected cutoff point, a sensitivity of 0.67 and a specificity of 0.75 were achieved. **Conclusions:** This highlights the system’s future potential for the early identification of patients at risk of readmission. For future implementation in clinical practice, an extensive clinical validation process will be required, along with the expansion of the database, which will likely contribute to improving the system’s robustness and generalization capacity.

## 1. Introduction

Chronic obstructive pulmonary disease (COPD) is characterized by the presence of chronic respiratory symptoms resulting from abnormalities in the airways and/or alveoli, causing persistent and often progressive airflow obstruction [1]. Currently, COPD is one of the most relevant public health problems, being one of the most common diseases worldwide and associated with high mortality and disability rates [1]. However, an increase in prevalence is expected in the coming years due, among other things, to exposure to toxins, tobacco smoke, and the aging of the population [2].

Acute exacerbations of COPD (AECOPD) are one of the most frequent events and generate great concern, since they imply a worsening of both the patient’s health status and quality of life, in addition to increasing the associated risk of mortality [3]. Furthermore, the most severe exacerbation episodes usually require hospital admission. Due to the high frequency of these events [1], a significant number of these patients will need to be readmitted for COPD during the weeks or months following the first hospitalization [4]. According to the AUDIPOC study, in which a total of 129 hospitals within the Spanish health system participated, the average readmission rate during the first 90 days is 37% [5]. On the other hand, the MAG-1 study, in which hospitals of the Catalan public health service participated, places the readmission rate at 90 days at 49% [6]. In the global context, some systematic reviews and meta-analyses were found in which readmission rates were analyzed. In the work of Ruan et al. [7], in which a total of twenty-eight studies were included, of which eight analyzed the 90-day readmission rate, a combined readmission rate of 31% was reported. On the other hand, in the meta-analysis conducted by Alqahtani et al. [8], which included a total of thirty-two studies, the 90-day readmission rate ranged from 17.5 to 39.0%. From a socioeconomic point of view, AECOPD admissions have an incredibly significant impact. In the systematic review conducted by Njoku et al. [9], which included a total of fifty-seven studies from thirty different countries, it was concluded that readmissions contribute greatly to the increase in total costs of COPD. More specifically, almost two-thirds of the total cost associated with exacerbations, which account for between 60 and 70% of the direct cost of COPD in Spain, are related to AECOPD episodes requiring admission [4,10].

To respond to this problem, various proposals have been put forward in recent years which, supported using various predictive models and scales, attempt to provide support in the management of patients with COPD. In the systematic review conducted by Bellou et al. [11] only thirty-six combined prognostic models of readmission and mortality were found, although none of them focused exclusively on readmission. Other initiatives explored risk factors for readmission, to facilitate the subsequent construction of predictive models. To date, most studies have only analyzed variables from the clinical and demographic spheres. Nevertheless, as reported in major systematic reviews [7,12], the heterogeneity of the studies is very high, suggesting that the existing evidence on risk factors for readmission due to AECOPD is limited, which may in some way be due to the wide heterogeneity of the disease.

In general, clinical practice can be understood as a continuous decision-making process. To facilitate physicians in making good decisions, it is increasingly common to develop decision support systems. Those driven using techniques from the field of artificial intelligence (AI) are known as intelligent decision support systems, and there are currently numerous applications in the clinical field [13,14,15,16,17,18,19,20,21,22,23,24,25,26,27,28,29,30,31,32,33,34,35,36,37,38,39,40,41,42,43,44,45]. In relation to COPD, multiple studies were found that use these tools to predict events such as mortality or deterioration of lung function, however, very few focus on predicting readmission. In the study made by Goto et al. [46], administrative data from over 1000 hospitals in Japan were used, selecting 44,929 patients and 420 variables to evaluate the performance of machine learning-based models compared to conventional statistical models for predicting 30-day readmissions. Two models were developed: one based on Lasso regression and the other on a deep neural network. The results showed better performance of the machine learning models compared to conventional predictive tools. On the other hand, in the study conducted by Liew et al. [47], data from the electronic medical records of 569 patients hospitalized for COPD at the National Taiwan University Hospital were used to develop a new predictive index for 30-day readmissions. The predictive model combined a new index, incorporating a total of 30 variables, with a neural network. The results reported superior performance compared to conventional statistical indices, such as the LACE index, with an area under the curve (AUC) of 0.76. Similarly, in the study by Wang et al. [48], a neural network-based model was used for predicting 30-day readmissions. To that end, data from 332 patients from eight hospitals in Houston, USA, were used, selecting four predictive variables. The results reported an approximate AUC of 0.77. In other research, such as the one carried out by Li et al. [49], other machine learning algorithms were used, specifically a decision tree-based approach with a Bayesian hyperparameter optimizer, to predict 30-day readmissions. The data were derived from the electronic medical records of 782 patients in a hospital in Macau, and seven statistically significant variables were selected using the Chi-Square test. The results reported an AUC of 0.75. In the study by Zhang et al. [50], a support vector machine approach was used for predicting 30-day readmissions. Data were collected from 1058 patients across 13 different hospitals in the Ningxia region of China. Eight variables, also determined to be statistically significant using the Chi-Square test, were included in the model. The best results reported an AUC of 0.88. In the study conducted by López et al. [51], a total of 5794 patients were included, of which 1893 had asthma and 3901 had COPD. Various machine learning models—Random Forest, Naïve Bayes, support vector machines, Gradient Tree Boosting, and a multilayer perceptron—were used to evaluate and compare their performance in predicting 30-day readmissions, utilizing a total of 60 clinical variables. The results showed an AUC above 0.83, with the best results obtained from support vector machines, achieving an AUC of 0.88. Conversely, in the study by Verma et al. [52], data on the physical activity of 16 patients were used to predict 30-day readmissions using four different machine learning algorithms: logistic regression, support vector machines, Random Forest, and a multilayer perceptron. The model based on logistic regression achieved the best performance. In general terms, a series of comments should be made about the different works. Firstly, it is important to highlight the methods used for variable selection, some of which are based on experience or the use of univariate statistical approaches. Moreover, most studies exclusively utilize variables from clinical and demographic domains. Additionally, all the studies mentioned focus on predicting short-term (30-day) readmissions and, in no case, consider predictions for a longer-term horizon (90 or 365 days). Furthermore, all of them use simple architectures, relying on a single machine learning algorithm. Beyond these studies, the proposal by Mohamed et al. [53] should be highlighted. In their study, the precision of various machine learning algorithms, including decision trees, neural networks, and support vector machines, was compared for predicting short and medium-term (30 and 90-day) readmissions. While short-term predictions (30 days) showed acceptable performance, with AUCs of 0.77, 0.75, and 0.73 for each algorithm, respectively, medium-term predictions (90 days) yielded more moderate results, with AUCs of 0.64, 0.62, and 0.61, respectively. As can be seen, there is a lack of studies in the literature addressing the availability of systems for medium-term (90-day) readmission prediction. Most proposals focus on short-term predictions. Proposing a broader prediction horizon provides clinicians with highly relevant information, which can enable the design of follow-up and continuous care strategies.

The development of specific tools that facilitate the identification of patients at higher risk of readmission would allow the design of strategies aimed at reducing the frequency of these events. These strategies could focus on providing more intensive care and closer monitoring by the medical team. In this way, the number of readmissions could be reduced, which would contribute to reducing the socioeconomic burden of the disease and improving both the health status and quality of life of patients. Therefore, this work proposes the design, development, and proof of concept of a new clinical intelligent decision support system applied to the prediction of hospital readmissions at 90 days in patients with AECOPD.

The main contributions of the article are as follows:

- Introduce, from a conceptual perspective, the architecture of a new intelligent decision support system to predict 90-day hospital readmissions in patients with AECOPD.
- Starting from an extensive database, select a reduced subset of variables suitable for predicting 90-day hospital readmissions in patients with AECOPD.
- Implement the designed system into a software artifact and demonstrate its applicability through a practical case study.

The work is divided into five sections. In Section 1, the framework of the proposed system in this research is defined. In Section 2, Section 2.1 presents the database used to build the system. Section 2.2 presents the conceptual design of the system. Following that, Section 2.3 discusses the implementation of the system through a software artifact. Section 3 presents a case study that exemplifies the system’s functionality as a proof of concept. Section 4 provides a discussion of the work. Finally, Section 5 presents the conclusions.

## 2. Materials and Methods

### 2.1. Database Usage

For the definition of the system, a database with a total of 593 patients was used, resulting from the combination of two cohorts from previous studies carried out by members of the research team [54,55]. Both cohorts included patients admitted for AECOPD to the Pulmonology department of either Álvaro Cunqueiro Hospital in Vigo or Lucus Augusti Hospital in Lugo, two public hospitals in Northwest Spain, which together serve a reference population of 600,000 inhabitants. Clinical staff and social workers were responsible for collecting all necessary information about each patient by reviewing the medical records and conducting numerous interviews with both patients and their respective caregivers. Subsequently, a follow-up was carried out for one year to record readmissions. To do this, the electronic medical records of each patient were reviewed at 30, 90, and 365 days after the index admission. In this case, only admissions at 90 days will be considered. Choosing a medium-term prediction horizon offers a broader and more clinically relevant approach. This period allows clinicians to design strategies for continuous and personalized follow-up of the patients. For more information on the data collection methodology, previous studies can be reviewed [54,55].

The database includes numerous variables from the clinical, demographic, and social spheres, which are summarized in Table 1, where the name and type of variable (categorical or numerical) are indicated, as well as a brief description of the variable. Categorical variables are binary, so they were coded by assigning the values 0 and 1. For example, in the variable “Smoking”, the value 0 was assigned to non-smoking patients and the value 1 to smoking patients. The rest of the variables were coded in a similar way.

In addition to these, 90-day readmissions are included, which acts as a label and as a dependent variable to predict.

Of the total number of patients, 460 patients are finally analyzed due to missing data. Of these, 20% of the patients are reserved for testing purposes, the rest will be used for training and system definition. It is important to note that the determination of the rule set will be made exclusively using the dataset reserved for training the learning models. The test set will be reserved solely for evaluating the model’s generalization ability.

### 2.2. Conceptual Design

**Figure 1:**
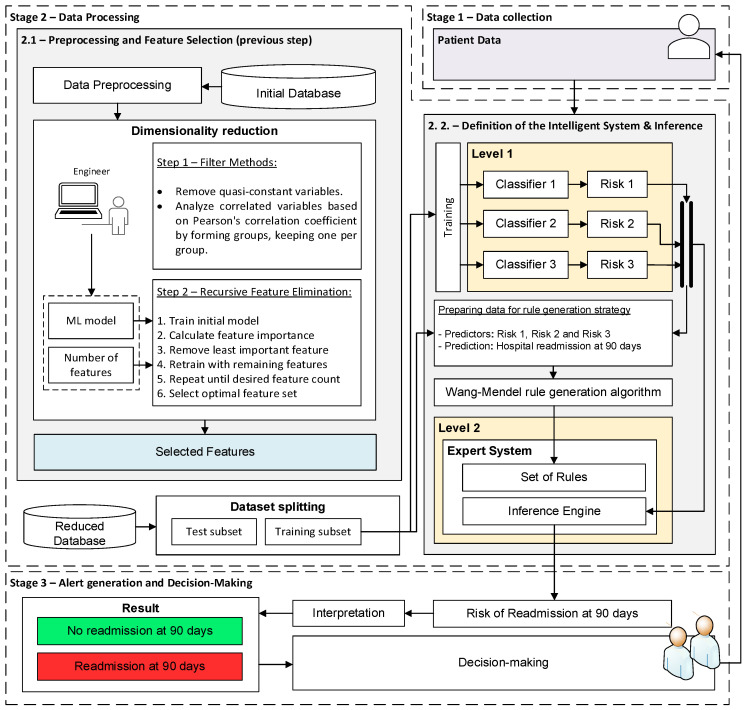
Flow diagram of the intelligent decision support system: The system is organized in three stages. Stage 1: Collection of patient data. Stage 2: Data processing, divided into two sublevels. In Stage 2.1, variable selection is performed in two steps: filter-type methods and recursive feature selection. In Stage 2.2, three ML models predict the risk of readmission, and an expert system based on fuzzy logic combines the predictions into a final output. Stage 3: Alert generation and support for clinical decision-making.

The flow chart of the intelligent system proposed in this work is presented in Figure 1. A detailed description of the different stages is presented below.

#### 2.2.1. Data Collection

As can be seen, firstly, patient data collection is carried out, which has been previously presented in Section 2.1. However, not all available variables are used, but a specific subset, including age, body mass index (BMI), pack-year index (PYI), % FEV_1_, % Eosinophils in blood, CAT score, number of positive cultures, number of hospitalizations in the previous year, days of stay, dyspnea according to the mMRC scale, number of basic activities with dependency and number of instrumental activities with dependency.

#### 2.2.2. Data Processing

After collecting and structuring the patient’s data, the system processes it. To do this, an intelligent system arranged in two levels is proposed. In the first level, three machine learning classification models are deployed, which run concurrently, obtaining at their output, in each case, a risk indicator. After this, in the second level, a fuzzy expert system of the Mamdani type is deployed, which acts as an aggregator of the risks obtained in the first level, obtaining at its output the *Risk of Readmission at 90 days*. Below is a description of the stages necessary for the definition of the system, from preprocessing and feature selection to inference:

- Stage 2.1 - Preprocessing and feature selection: First, the patient variables are preprocessed. For numerical variables, a min-max normalization is applied, which adjusts their values to the range [0,1]. Categorical variables are converted to numerical values: in the case of binary categorical variables, a “1” is used to indicate the presence of a given condition or characteristic, and a “0” otherwise. Once the preprocessing is completed, feature selection [56] is performed, with the aim of finding an optimal subset of features that will facilitate the future implementation and transfer of the system in clinical practice and ensure its acceptance by professionals. This feature selection process is carried out in two sequential steps:
  ○ Filter-type feature selection methods: First, the variables that are practically constant are eliminated, that is, those that present identical values in 98% of the cases. Then, the variables that are correlated are analyzed, grouping those whose Pearson correlation coefficient is greater than 0.8, and keeping only one of them in each group.
  ○ Recursive Feature Elimination: The Recursive Feature Elimination (RFE) method [56,57] is used, using a Random Forest model. RFE method can be categorized as a hybrid method since it considers characteristics of both wrapper and embedded methods [56]. RFE, in essence, allows selecting an optimal subset of features iteratively, eliminating the least important ones, and retraining the model until reaching the desired set. After performing various tests, a final set with 12 predictive variables was determined, including age, body mass index (BMI), pack-year index (PYI), % FEV_1_, %Eosinophils, CAT score, number of positive cultures, number of hospitalizations in the previous year, days of stay, dyspnea according to the mMRC scale, number of basic activities with dependence and number of instrumental activities with dependence.
- Stage 2.2 - Definition of the intelligent system and inference: Once the variables with the highest predictive power have been selected, they are processed by the system. To do this, as mentioned, a two-level system is deployed. At the first level, three machine learning algorithms are presented that run concurrently, and to which the twelve patient variables are provided. More specifically, three machine learning algorithms have been selected following Peter Flach’s recommendations [58]: Random Forest, Naïve Bayes, and Multilayer Perceptron. At the output of each of these, a risk indicator is obtained, the risks R_1_, R_2_, and R_3_. After that, at the second level, a fuzzy expert system of the Mamdani type is deployed [59,60,61,62], which allows the risks obtained at the first level to be added and grouped, obtaining the *Risk of Readmission at 90 days*. In general, one of the most complex tasks in the design and development of an expert system is the definition of its knowledge base. For this reason, in this work, the use of the Wang–Mendel automatic rule generation algorithm [63,64] has been chosen so that, starting from a new dataset formed by risks R_1_, R_2_, and R_3_ obtained for each patient in the training dataset, it is possible to generate the knowledge base of the expert system. The test set was not used under any circumstances to determine the knowledge base of the expert system. It was exclusively reserved for the model’s subsequent evaluation. Once the system architecture has been defined and given the data of a new patient, it is possible to carry out its analysis, obtaining the associated *Risk of Readmission at 90 days*.

#### 2.2.3. Alert Generation and Decision-Making

Once the patient data have been analyzed, the *Risk of Readmission at 90 days* is obtained. In this last stage, its interpretation is carried out, obtaining the associated label (readmission vs. no readmission at 90 days). Based on this, physicians can take the most appropriate measures for patient care.

### 2.3. System Implementation

This section details the implementation of the intelligent system presented in the previous section through a software artifact. For this purpose, the Python environment (version 3.11.7) has been mainly used, with the Pandas, Feature Engine, Scikit Learn, Skfuzzy, and Tkinter libraries. In addition, MATLAB (version R2024a, Natick, MA, USA) has been used, together with the Fuzzy Logic Toolbox, for the automatic rule generation strategy with the Wang–Mendel algorithm.

The equipment used for the implementation of the system consists of an Apple M2 Pro processor, with an integrated 16-core GPU and 16 GB of RAM.

**Figure 2:**
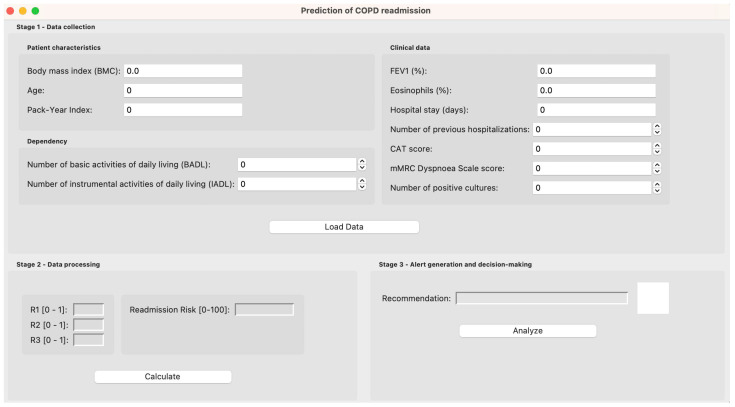
Screenshot of the main system interface.

Figure 2 presents a screenshot of the system’s main interface.

#### 2.3.1. Data Collection

First, the patient’s data are entered into the application using the forms that can be seen in Figure 2. It is recommended that the medical team verify the correct entry of the data to ensure the accuracy of the predictions.

#### 2.3.2. Data Processing

Once the data have been loaded, the processing is carried out. To do this, the user will go to the corresponding panel in the application, as can be seen in Figure 2.

##### Preprocessing and Feature Selection

As reflected in Figure 1, prior to defining the intelligent system, an initial selection of characteristics is carried out, with the aim of finding a subset of the variables with the greatest predictive power.

As described, the feature selection process is carried out in two steps. First, filtering techniques are applied. After this, RFE is applied to the remaining variables, supported using the Random Forest model with 100 decision trees and for which the following specific parameters were defined: use of the Gini criterion to evaluate the quality of the divisions, with no maximum depth limit and a requirement of a minimum of 2 samples to divide a node. This entire selection process has been carried out using the Feature Engine library.

**Figure 3:**
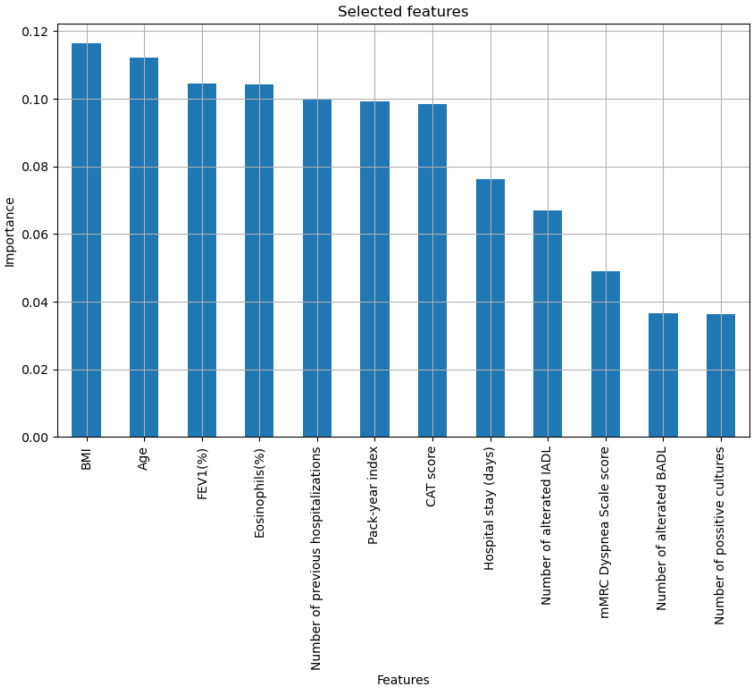
Selected variables ranked by importance after the variable selection process using RFE.

After carrying out various tests, a subset with 12 variables was determined, as can be seen in Figure 3, ordered by importance, according to the associated information gain.

##### Definition of the Intelligent System and Inference

Once the twelve predictor variables have been selected, the intelligent system is defined. To do this, as mentioned, an assembly strategy arranged on two levels is used.

Using the twelve variables selected, three machine learning models are trained at the first level, obtaining the risks R_1_, R_2_, and R_3_, which are associated with the risk of the patient being readmitted within 90 days according to each of the models. These risks vary in the range between 0 and 1.

A summary of the training strategy for each of the models is presented in Table 2. The training of the models has been completed using the Scikit-Learn library.

After training the models, a new dataset is determined by calculating the values of R_1_, R_2_, and R_3_ for each patient in the training set, which act as predictor variables, and the variable to be predicted, which is the 90-day readmission (expressed as 0 or 1). This dataset is essential in the second level of the assembly strategy. As mentioned, a fuzzy expert system with a Mamdani-type inference engine is deployed at this level. Based on this new dataset, the Wang–Mendel automatic rule generation algorithm is used to determine the knowledge base. A summary of the steps of the Wang–Mendel automatic rule generation algorithm [63] is presented in Table 3, as well as specific comments on the application in this work.

##### Analysis of Results in the Test Set and Search of the Optimal Threshold

In essence, the proposed system, understood as the assembly just defined, behaves as a binary classifier, which allows us to distinguish between patients who will be readmitted after 90 days and those who will not. The generalization capacity of the system on the set of patients reserved for testing is then analyzed. These patients were not used in the process of building the system.

**Figure 4:**
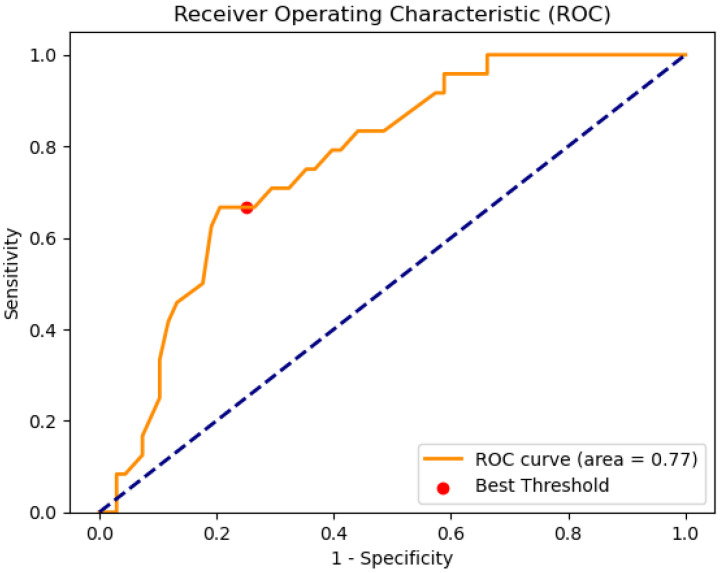
ROC curve on the test set. A red point is highlighted, related to the point that maximizes the value of the Matthews correlation coefficient.

Figure 4 presents the ROC curve, with an AUC value of 0.77.

As mentioned, the system behaves as a binary classifier. To determine one label or another (readmission vs. no readmission) it is necessary to determine a threshold value to interpret the *Risk of Readmission at 90 days* (the output of the expert system). In this case, the use of a graphical optimization process on the test set based on the Matthews correlation coefficient (Mcc) [65,66,67] is chosen, similar to that used by Casal-Guisande et al. in other works [30,31,34,64]. This is a coefficient that behaves especially well in situations where there is imbalance in the data. The expression for Mcc is presented in equation 1, where *TN* = True Negatives, *FN* = False Negatives, *TP* = True Positives, and *FP* = False Positives.

(1) $$Mcc=\frac{TN\cdot TP-FN\cdot FP}{\sqrt{\left(TP+FP)\cdot (TP+FN)\cdot (TN+FP)\cdot (TN+FN\right)}}$$

**Figure 5:**
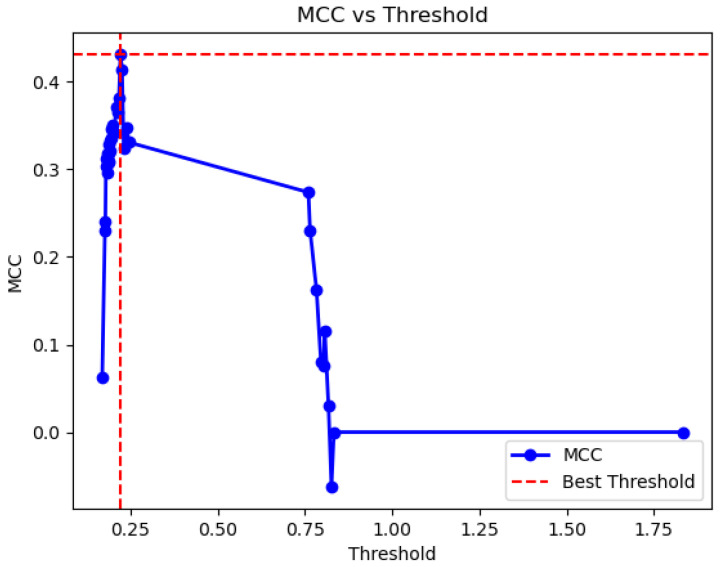
Determination of the optimal cutoff point based on the Matthews correlation coefficient.

**Figure 6:**
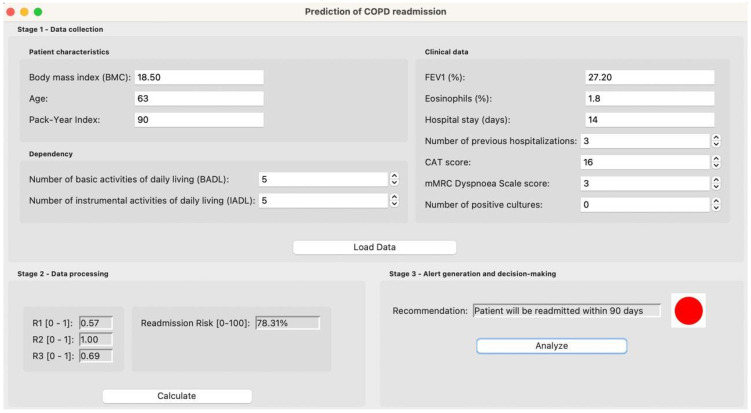
Capture of the results obtained in the case study.

Figure 5 presents the graphical optimization process, obtaining an optimal threshold of 0.22, with an associated Mcc of 0.43. In Figure 6, this point is highlighted in red on the ROC curve. For the selected cutoff point, a sensitivity of 0.67 and a specificity of 0.75 were obtained.

#### 2.3.3. Alert Generation and Decision-Making

After processing the patient data, the system will suggest one of the following labels: “*The patient will be readmitted within 90 days*” or “*The patient will not be readmitted within 90 days*”. This information is also displayed in the application, as can be seen in Figure 2.

Considering the system’s recommendations, the medical team will be able to make the best decisions for the care and monitoring of patients who have a higher risk of short-term readmission.

## 3. Case Study

A case study of the proposed system is presented below, which seeks to exemplify its operation with a simple case, and which can be understood as a proof of concept. A patient not contemplated in the construction of the system will be used, one of those reserved in the test set. It is also important to point out that the system is in the early stages of development, and that in the future it will be necessary to address intensive clinical validation in real environments.

### 3.1. Initial Data Collection

Table 4 presents the data of the patient used in this case study. The patient is a 63-year-old male diagnosed with COPD who was admitted for AECOPD. He has a BMI of 18.5, which is within a healthy range. He has a high smoking habit with a PYI of 90 packs/year and impaired lung function, presenting an %FEV_1_ of 27.20% in the last spirometry, which means he has a very severe obstructive pattern. The percentage of eosinophils (1.8%) is within normal ranges, and he did not present any positive cultures during the year prior to admission. He obtained a score of 16 points on the CAT questionnaire, which indicates a medium impact of the disease. On the other hand, he obtained a score of 3 points on the mMRC dyspnea scale, which is associated with severe dyspnea. The patient was admitted for 14 days and had already been admitted during the previous year on three occasions. In addition, the patient presents a high dependency with five BADL and five AIDL affected.

To compare the prediction with what happened, it is important to note that the patient was readmitted before 90 days.

### 3.2. Data Processing

Once the data have been entered into the application, as can be seen in Figure 6, it is processed by the system.

Based on the patient data, a *Risk of Readmission at 90 days* of 78.31% is obtained, as can be seen in Figure 6.

### 3.3. Alert Generation and Decision Making

Based on the *Risk of Readmission at 90 days*, and considering the previously determined threshold, the label “*The patient will be readmitted within 90 days*” is determined, as can be seen in Figure 6.

This prediction matches what actually happened. Based on this information, specialists could have monitored the patient more closely, redirecting treatments, which could have meant that the patient would not have been readmitted so soon.

## 4. Discussion

COPD is a complex pathology characterized by recurrent exacerbations, which, in the most severe cases, require hospitalization. These exacerbations not only negatively affect the patient’s quality of life but also represent a significant challenge for health systems due to the high care burden and the resources they consume. In recent years, various state-of-the-art proposals have been developed based on the use of artificial intelligence techniques, with the aim of facilitating the management of patients with COPD. However, predicting readmission remains a challenge, and there are hardly any proposals in the state of the art. In addition, most of the proposals in the state of the art focused on predicting short-term readmissions (30 days) [46,47,48,49,50,51,52], which helps identify the most severe patients but prevents comprehensive follow-up and efficient planning and management of chronic diseases such as COPD. To address this problem, this work has proposed a new intelligent clinical decision support system applied to predicting hospital readmissions at 90 days after an exacerbation episode in patients with COPD.

Unlike other proposals and approaches commonly used in the state of the art, the proposed system introduces a new architecture that integrates machine learning techniques together with symbolic inference techniques arranged in two levels. At the first level, a set of machine learning algorithms are deployed that run concurrently, generating at their output different risk metrics related to the danger associated with the patient being readmitted before 90 days; after this, at the second level, the outputs of the machine learning algorithms of the first level are integrated through an expert system that uses a fuzzy inference engine, whose knowledge base has been determined using Wang–Mendel’s automatic rule generation algorithm.

From a global perspective, the proposed architecture can be understood as an ensemble approach that integrates and weights the predictions of multiple heterogeneous algorithms, which contrasts with more conventional ensemble strategies, which typically employ multiple instances of the same model, as occurs in Random Forest or Gradient Boosting. While traditional approaches use repeated versions of the same type of algorithm, in this case, decision trees, to improve robustness, the proposed system combines algorithms of different natures, allowing the unique strengths of each to be leveraged. In this case, the model acts similarly to an ensemble but uses a Fuzzy Expert System as an aggregator, allowing for a more flexible and robust combination of predictions. Additionally, the proposed ensemble structure is characterized by its high versatility, as it allows for the interchangeability of the first-level engines. This means that the base algorithms can be easily replaced or updated with minimal adjustments, making them adaptable to different domains, data types, and specific constraints. By being trained independently, each model helps reduce overall computational complexity while ensuring a scalable and efficient approach. To exemplify the functioning of the system, three algorithms representative of the main machine learning families have been selected in this study, although the approach is flexible, and other models with different hyperparameter configurations could be used. The advantage of using diverse algorithms lies in the ability to capture different patterns and relationships in the data, which enriches the precision and adaptability of the model. In addition, the integration of an expert system that allows combining the outputs of the machine learning algorithms represents an innovative approach. Expert systems are one of the main tools of deductive reasoning in artificial intelligence, and they stand out for their ability to formalize and diversify knowledge. In this case, the rules that make up the knowledge base of the expert system do not represent knowledge in the traditional sense. These rules have been generated using the Wang–Mendel algorithm, using a dataset derived from the original that includes the predictions of the machine learning models and the labels associated with the readmission. What is particular about this approach is that the rule priors do not reflect traditional medical concepts, such as a symptom or a clinical variable, but are based on the risks calculated by the algorithms defined in this work. Therefore, the rule priors do not correspond to common medical jargon, which explains why they do not determine clinical knowledge in the conventional sense. This process generates a knowledge base tailored to the algorithm outputs, allowing the expert system to aggregate the predictions in a coherent and comprehensible way. In this way, the system not only integrates the information coming from the different algorithms effectively but also provides a clear and traceable explanation of how each decision has been reached, increasing confidence in the predictions and facilitating their application in clinical practice.

Another aspect worth highlighting is the initial dimensionality reduction, a crucial step to facilitate the future transfer of the system to the healthcare field once validated. This reduction allows for defining a limited set of easily accessible variables, which can be collected in a brief clinical interview, which in turn favors the acceptance of the system by healthcare professionals. Unlike the studies existing in the current literature, which use conventional statistical methods for variable selection, in this study, a two-phase variable selection strategy has been implemented. In the first phase, simple techniques, commonly categorized as “filter methods”, were used, which allowed discarding constant or almost constant variables, and in the case of highly correlated variables, it was decided to retain only one of them per group. Subsequently, the Recursive Feature Elimination (RFE) technique was applied, which can be classified as a hybrid variable selection technique. After various experiments, a final subset of 12 variables belonging to the clinical, demographic, and social spheres was determined.

It is particularly relevant to highlight the inclusion of social factors, which are often ignored in the management of patients with COPD. In recent years, awareness has increased about the importance of the social determinants of health in these patients, and this perspective has been incorporated in this work, which guarantees a more comprehensive characterization. By including variables related to dependency and the social environment, the proposed system can identify those patients who are more fragile and at greater risk of readmission, contributing to a more precise and personalized approach to their management.

The implementation of intelligent decision support systems, such as the one proposed in this work, has the potential to significantly transform the way in which patients with COPD are managed, especially those at risk of readmission. The ability of the system to facilitate early detection of patients at higher risk of readmission before 90 days allows physicians to anticipate possible adverse outcomes, improving clinical decision-making. By more accurately identifying the most fragile patients, it is possible to proactively adjust treatments, prioritizing those that maximize quality of life and minimize complications. Furthermore, the unification and systematization of clinical decisions is a crucial aspect that this type of system offers. In the management of patients with COPD, medical decisions can vary significantly between professionals due to the nature of the disease. By being based on objective data and automated processes, the proposed system allows for reducing this variability, standardizing patient management, and ensuring that all patients receive consistent, evidence-based care. This is especially relevant in environments with high staff turnover or where multidisciplinary teams are involved, as it ensures that a consistent and optimal approach to treatment is maintained.

In conclusion, this system not only represents an advanced tool for predicting hospital readmissions but also opens the door to a more equitable and effective management of COPD patients. By allowing for earlier care planning and more unified decision-making, the system has the potential to significantly improve both clinical outcomes and patient experience in healthcare services.

### 4.1. Relevance of the Proposal

In order to highlight the contributions made in this article, Table 5 provides a comparison of the proposed system with other existing works in state of the art based on five criteria: reasoning (understood as the system’s ability to use symbolic reasoning); scalability (understood as the system’s ability to modify its calculation engine); efficiency (understood as the system’s ability to manage uncertainty); data dependence; and medium-term prediction capability.

Analyzing Table 5 reveals that all the works in the state of the art rely exclusively on machine learning techniques. This contrasts with the proposed architecture, which combines learning-based approaches with symbolic inference methods, organized into a two-level system. This architecture, along with the incorporation of the Wang–Mendel automatic rule generation algorithm, provides the model with remarkable scalability, enabling the integration of new calculation engines at the first level and allowing for straightforward adjustments. Furthermore, the ability to make medium-term predictions represents a clear differentiating factor, which has been scarcely explored in the literature, as evidenced by the review conducted.

### 4.2. Limitations and Future Lines of Development

Among the main limitations of this study is the size of the database used. Although it includes 593 carefully characterized patients, it could be considered limited in the context of machine learning. This sample size has influenced the choice of models employed in the system’s design, ruling out the possibility of implementing deep learning approaches, which typically require larger datasets to ensure effective training and avoid overfitting issues.

It is important to note that the database originates from the merger of two cohorts from previous studies. While this provides diversity and robustness to the data, it also implies that the database was not specifically designed for the objectives of the present work, potentially introducing limitations inherent to the original characteristics of these cohorts. This highlights the importance of considering potential biases in data representativeness and their impact on the results obtained.

From a technical perspective, optimizing the rule selection process in the Wang–Mendel algorithm is also necessary, as it could be associated with the loss of relevant information during the automatic rule generation process. Improving this aspect would strengthen the robustness of the system and ensure the maximum utilization of the database’s features.

In future work, it will be essential to conduct thorough clinical validation using independent and external datasets to adapt the system to daily clinical practice and evaluate its performance in real clinical scenarios. Additionally, expanding the database to include a larger number of cases will be a priority, as this will enhance the system’s robustness and improve its generalization capacity for the results obtained.

Despite these limitations, the use of a well-characterized database and the implementation of a robust methodological design contribute to the validity of the results obtained and provide a solid starting point for future research in this field.

## 5. Conclusions

This work presents an intelligent clinical decision support system designed to predict the risk of hospital readmission within 90 days for patients with COPD following an acute exacerbation. This is the first system focused on a 90-day prediction horizon, offering the advantage of broader management and the ability to provide a continuous and personalized follow-up of the patients at risk. Unlike other proposals described in the literature, the system employs a unique architecture that combines the predictions of three machine learning algorithms —Random Forest, Naïve Bayes, and Multilayer Perceptron—using an expert system supported by a fuzzy inference engine. The knowledge base of the system has been generated using the Wang–Mendel automatic rule generation algorithm. To reduce the dimension of the problem and enhance the system’s applicability to future clinical practice, a novel approach for variable selection was adopted, moving away from conventional statistical methods. This approach utilizes filter-type methods and recursive feature selection supported by the Random Forest algorithm. As a result, a set of 12 variables was used to construct the intelligent system. Additionally, in contrast to other studies, the system incorporates variables from the social dimension, which enriches the model and introduces a new dimension to the management of readmissions in COPD patients.

The operation of the system has been illustrated by a case study, which has allowed it to show its future viability in the clinical context and its ease of use. Although the results obtained in the test subset are promising, with an AUC close to 0.8, a sensibility of 0.67, and a specificity of 0.75 at the selected cut-off point, it is important to note that the system is still in an early stage of development, so more extensive validation is necessary before its application in real clinical environments.

Among the limitations of this study, it is worth highlighting that the size of the database used is relatively small, which could limit the ability to generalize the results. Also, from a technical perspective, it is crucial to improve the rule selection process in the Wang–Mendel algorithm to avoid the possible loss of relevant information in the generation of rules.

In future work, it will be essential to carry out thorough clinical validation to adapt the system to daily practice and evaluate its performance in real clinical scenarios. In addition, expanding the database to include more cases will be a priority to increase robustness and improve the system’s capacity to generalize its results.

## Figures and Tables

**Table 1:** Summary of the variables.

Variable	Type	Comments
Sex	Categorical	Male (1)/Female (0)
Age	Numerical	-
BMI (kg/m^2^)	Numerical	-
Active smoker	Categorical	Yes (1)/No (0)
Drug abuse	Categorical	Yes (1)/No (0)
High ingestion of alcohol	Categorical	Yes (1)/No (0)
Pack-year index (PYI)	Numerical	The product is obtained by multiplying the number of cigarettes smoked per day by the number of years the person has been smoking
Number of previous hospitalizations in the previous year	Numerical	-
Hospital stay (days)	Numerical	Number of days of the current hospital stay
Number of positive sputum culture within the previous year	Numerical	-
Pneumococcal vaccination	Categorical	Yes (1)/No (0)
Influenza vaccination previous year	Categorical	Yes (1)/No (0)
% Eosinophils	Numerical	-
% FEV_1_	Numerical	-
Dyspnea mMRC	Numerical	-
CAT score	Numerical	-
Anemia	Categorical	Yes (1)/No (0)
Cardiovascular disease	Categorical	Yes (1)/No (0)
Obstructive sleep apnea	Categorical	Yes (1)/No (0)
Depression and/or anxiety	Categorical	Yes (1)/No (0)
Hypertension	Categorical	Yes (1)/No (0)
Arteriopathy	Categorical	Yes (1)/No (0)
Diabetes mellitus	Categorical	Yes (1)/No (0)
Cancer	Categorical	Yes (1)/No (0)
Continuous home oxygen therapy	Categorical	Yes (1)/No (0)
Home non-invasive ventilation	Categorical	Yes (1)/No (0)
Inhaled corticosteroids at discharge	Categorical	Yes (1)/No (0)
Place of residence	Categorical	Urban (1)/Rural (0)
Monthly income > EUR 800	Categorical	Yes (1)/No (0)
Employment status	Categorical	Active (0)/Retired (1)
Housing situation	Categorical	No property (0)/Owns property (1)
Living alone	Categorical	Yes (1)/No (0)
Sleeps alone	Categorical	Yes (1)/No (0)
Caretaker available	Categorical	Yes (1)/No (0)
Previous use of social services resources in the last 12 months	Categorical	Yes (1)/No (0)
Social relationships	Categorical	Neighbors, friends (1)/None or only with family (0)
Drives car	Categorical	Yes (1)/No (0)
Number of basic activities of daily living with dependency (BADL)	Numerical	The number of basic activities for which the individual is dependent is recorded. There are 5 (eating, dressing, taking a bath, going to the toilet, moving) so they vary in a range from 0 to 5.
Number of instrumental activities of daily living with dependency (IADL)	Numerical	The number of instrumental activities for which the individual is dependent is recorded. There are 8 (help with food preparation, house cleaning, laundry, telephone use, shopping, financial management, transport and medication), so they vary in range from 0 to 8.

**Table 2:** Training strategy overview.

Mode	Parameters
Random Forest	100 decision trees, uses the Gini criterion to evaluate the quality of the splits, with no maximum depth limit, and requires a minimum of 2 samples to split a node.
Gaussian Naive Bayes	It uses a Gaussian probability distribution, with a variance smoothing of 1 × 10^−9^ to avoid divisions by zero.
Multilayer perceptron	1 hidden layer with 9 neurons, uses the Logistic activation function, the Adam optimizer, an L2 regularization with a value of 0.0001, a constant learning rate, and a maximum of 1000 epochs.

**Table 3:** Strategy for automatic rule generation using the Wang–Mendel algorithm [63].

Steps	Description	
Previous considerations	Before applying the Wang–Mendel algorithm it is required: Define the form of the membership functions: For the antecedents (R_1_, R_2_, and R_3_) and the consequent (Readmission at 90 days) triangular membership functions are used. Definition of the number of sections of the membership function in each variable: A parameter N must be defined, which can take as value the natural numbers, related to the number of sections of the membership function of each variable. The number of sections is calculated using the expression 2N + 1, so that the presence of a central section is always guaranteed. In the case of the antecedents, N = 2 was set, which results in 5 sections in each case. In the consequent, N = 1 was set, which results in 3 sections. In this last case, the output is binary, so the central section will not be used.	
1—Division of input and output spaces into fuzzy regions	Taking into account the previous considerations, in this step the division of the input spaces (antecedents: R_1_, R_2_, and R_3_) and output (consequent: readmission at 90 days) is carried out. As proposed in the original paper by Wang–Mendel, the overlap between the adjacent sections of the membership functions is considered, as can be observed below. In the case of the consequents, the central section of the membership function was suppressed, since it can only take two values (0 or 1).	
	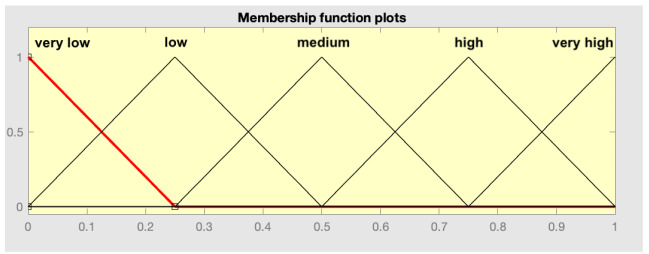	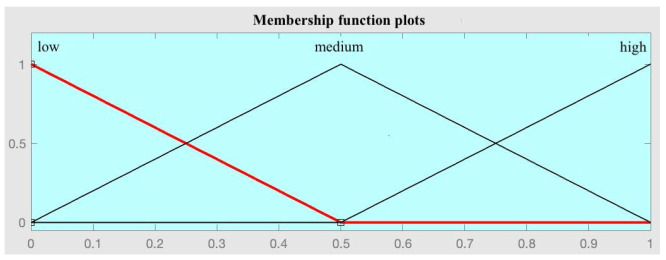
	Antecedent space division	Consequent space division
2—Fuzzy rule generation	Taking the determined fuzzy spaces as a reference, we proceed to the generation of rules. For each patient in the dataset used and for each variable, the degree of membership in each section of the membership functions is calculated. After this, for each patient, each variable is assigned to the section that has the highest degree of membership, giving rise to one rule per patient. Given that the set reserved for training, in this case for the generation of the set of rules, had a total of 368 patients, 368 rules are obtained.	
3—Assign a grade to each rule to resolve conflicts	In the set of rules generated in the previous step, there may be cases where there are rules that, having the same antecedents, have different consequents. Following the recommendations of the original Wang–Mendel paper, a coefficient associated with each rule is determined, understood as the product of the degrees of belonging of the patient that gave rise to the rule. After this, in case of conflict, the rule with the highest value in this coefficient is maintained, discarding the rest. After this process, 50 rules remain, concluding the automatic rule generation process.	
4—Construction of a combined fuzzy knowledge base	Although it does not apply in this case, after the automatic rule generation process it would be possible to complement the set with new rules proposed by experts, applying, in case of conflicts between rules, an approach based on coefficients like the one already described.	
5—Inference	Taking the determined set of rules, and given data on new patients, it is possible to obtain the associated risk indicator, the *Risk of Readmission at 90 days*.	

**Table 4:** Patient data.

Variable	Value
Age	63
BMI	18.50
PYI	90
% FEV_1_	27.20
% EOS	1.8
Number of positive cultures	0
CAT	16
Dyspnea mMRC	3
Days of stay	14
Number of hospitalizations in the previous year	3
Number of ADLs with dependency	5
Number of AIVD with dependency	5

**Table 5:** Benchmarking. A comparison of the proposed system with the state of the art is made based on 5 criteria (reasoning, scalability, efficiency, data dependence, and medium-term prediction). For each of the studies in the state of the art, a symbol is included in relation to the present work. The symbol “-” indicates that the work in the state of the art has inferior characteristics compared to our work according to the criterion in question. The symbol “=“ indicates that the work in the state of the art has similar characteristics to those of the present work.

	Reasoning	Scalability	Efficiency	Data Dependence	Medium-Term Prediction Capability
Goto et al. [46]	The system uses statistical inference.	The system is not scalable.	The authors use Lasso regression and deep learning for their analysis. They employ a probabilistic approach for managing uncertainty.	The system depends completely on data.	Short-term prediction (30 days).
	-	-	**=**	=	-
Liew et al. [47]	The system uses statistical inference.	The system is not scalable.	The authors use neural networks for their analysis. They employ a probabilistic approach for managing uncertainty.	The system depends completely on data.	Short-term prediction (30 days).
	-	-	**=**	=	-
Wang et al. [48]	The system uses statistical inference.	The system is not scalable.	The authors use neural networks for their analysis. They use a probabilistic approach for managing uncertainty.	The system depends completely on data.	Short-term prediction (30 days).
	-	-	**=**	=	-
Li et al. [49]	The system uses statistical inference.	The system is not scalable.	The authors use decision trees for their analysis. They employ a probabilistic approach for managing uncertainty.	The system depends completely on data.	Short-term prediction (30 days).
	-	-	**=**	=	-
Zhang et al. [50]	The system uses statistical inference.	The system is not scalable.	The authors use support vector machines for their analysis. They employ a probabilistic approach for managing uncertainty.	The system depends completely on data.	Short-term prediction (30 days).
	-	-	**=**	=	-
Lopez et al. [51]	The system uses statistical inference.	The system is not scalable.	The authors use Random Forest, Naïve Bayes, support vector machines, Gradient Boosting Decision Trees, and multilayer perceptron for their analysis. They employ a probabilistic approach for managing uncertainty.	The system depends completely on data.	Short-term prediction (30 days).
	-	-	**=**	=	-
Verma et al. [52]	The system uses statistical inference.	The system is not scalable.	The authors use logistic regression, Random Forest, support vector machines, and multilayer perceptron for their analysis. They employ a probabilistic approach for managing uncertainty.	The system depends completely on data.	Short-term prediction (30 days).
	-	-	**=**	=	-
Mohamed et al. [53]	The system uses statistical inference.	The system is not scalable.	The authors use decision trees, neural networks, and support vector machines for their analysis. They employ a probabilistic approach for managing uncertainty.	The system depends completely on data.	Short- and medium-term prediction (30 and 90 days).
	-	-	**=**	=	=
Our proposal	The system uses statistical and symbolical inference.	The system is scalable; it is possible to modify the calculation engines.	The system uses three machine learning algorithms that run concurrently, and their outputs are aggregated in a fuzzy logic expert system. This allows for the handling of uncertainty from both probabilistic and non-probabilistic approaches.	The system depends completely on data.	Medium-term prediction (90 days).

## Data Availability

Dataset available on request from the authors.

## References

[1] Agustí A., Celli B.R., Criner G.J., Halpin D., Anzueto A., Barnes P., Bourbeau J., Han M.L.K., Martinez F.J., Montes de Oca M. (2023). Global Initiative for Chronic Obstructive Lung Disease 2023 Report: GOLD Executive Summary. Arch. Bronconeumol..

[2] Fernández-Villar A., Fernández-García S., Represas-Represas C. (2020). El Componente Social de La Enfermedad Pulmonar Obstructiva Crónica: ¿Un Rasgo Tratable de La Enfermedad?. Arch. Bronconeumol..

[3] Miravitlles M., Soler-Cataluña J.J., Calle M., Molina J., Almagro P., Quintano J.A., Riesco J.A., Trigueros J.A., Piñera P., Simón A. (2014). Guía Española de La EPOC (GesEPOC). Actualización 2014. Arch. Bronconeumol..

[4] Fernández García S. (2019). Factores Sociosanitarios Relacionados Con El Pronóstico y Consumo de Recursos Sanitarios En Pacientes Hospitalizados Por Una Agudización de Enfermedad Pulmonar Obstructiva Crónica. Ph.D. Thesis.

[5] Pozo-Rodríguez F., López-Campos J.L., Álvarez-Martínez C.J., Castro-Acosta A., Agüero R., Hueto J., Hernández-Hernández J., Barrón M., Abraira V., Forte A. (2012). Clinical Audit of COPD Patients Requiring Hospital Admissions in Spain: AUDIPOC Study. PLoS ONE.

[6] Escarrabill J., Torrente E., Esquinas C., Hernández C., Monsó E., Freixas M., Almagro P., Tresserras R. (2015). Auditoría Clínica de Los Pacientes Que Ingresan En El Hospital Por Agudización de EPOC. Estudio MAG-1. Arch. Bronconeumol..

[7] Ruan H., Zhao H., Wang J., Zhang H., Li J. (2023). All-Cause Readmission Rate and Risk Factors of 30- and 90-Day after Discharge in Patients with Chronic Obstructive Pulmonary Disease: A Systematic Review and Meta-Analysis. Ther. Adv. Respir. Dis..

[8] Alqahtani J.S., Njoku C.M., Bereznicki B., Wimmer B.C., Peterson G.M., Kinsman L., Aldabayan Y.S., Alrajeh A.M., Aldhahir A.M., Mandal S. (2020). Risk Factors for All-Cause Hospital Readmission Following Exacerbation of COPD: A Systematic Review and Meta-Analysis. Eur. Respir. Rev..

[9] Njoku C.M., Alqahtani J.S., Wimmer B.C., Peterson G.M., Kinsman L., Hurst J.R., Bereznicki B.J. (2020). Risk Factors and Associated Outcomes of Hospital Readmission in COPD: A Systematic Review. Respir. Med..

[10] Miravitlles M., García-Polo C., Domenech A., Villegas G., Conget F., De La Roza C. (2013). Clinical Outcomes and Cost Analysis of Exacerbations in Chronic Obstructive Pulmonary Disease. Lung.

[11] Bellou V., Belbasis L., Konstantinidis A.K., Tzoulaki I., Evangelou E. (2019). Prognostic Models for Outcome Prediction in Patients with Chronic Obstructive Pulmonary Disease: Systematic Review and Critical Appraisal. BMJ.

[12] Chow R., So O.W., Im J.H.B., Chapman K.R., Orchanian-Cheff A., Gershon A.S., Wu R. (2023). Predictors of Readmission, for Patients with Chronic Obstructive Pulmonary Disease (COPD)—A Systematic Review. Int. J. COPD.

[13] Casal-Guisande M., Represas-Represas C., Golpe R., Fernández-García A., González-Montaos A., Comesaña-Campos A., Ruano-Raviña A., Fernández-Villar A. (2024). Clinical and Social Characterization of Patients Hospitalized for COPD Exacerbation Using Machine Learning Tools. Arch. Bronconeumol..

[14] Wang L., Chen X., Zhang L., Li L., Huang Y., Sun Y., Yuan X. (2023). Artificial Intelligence in Clinical Decision Support Systems for Oncology. Int. J. Sci..

[15] Niraula D., Sun W., Jin J., Dinov I.D., Cuneo K., Jamaluddin J., Matuszak M.M., Luo Y., Lawrence T.S., Jolly S. (2023). A Clinical Decision Support System for AI-Assisted Decision-Making in Response-Adaptive Radiotherapy (ARCliDS). Sci. Rep..

[16] Naiseh M., Al-Thani D., Jiang N., Ali R. (2023). How the Different Explanation Classes Impact Trust Calibration: The Case of Clinical Decision Support Systems. Int. J. Hum. Comput. Stud..

[17] Lu S.C., Swisher C.L., Chung C., Jaffray D., Sidey-Gibbons C. (2023). On the Importance of Interpretable Machine Learning Predictions to Inform Clinical Decision Making in Oncology. Front. Oncol..

[18] Hager P., Jungmann F., Holland R., Bhagat K., Hubrecht I., Knauer M., Vielhauer J., Makowski M., Braren R., Kaissis G. (2024). Evaluation and Mitigation of the Limitations of Large Language Models in Clinical Decision-Making. Nat. Med..

[19] Shafi I., Din S., Farooq S., de la Torre Díez I., Breñosa J., Martínez Espinosa J.C., Ashraf I. (2024). Design and Development of Patient Health Tracking, Monitoring and Big Data Storage Using Internet of Things and Real Time Cloud Computing. PLoS ONE.

[20] Shafi I., Din S., Khan A., Díez I.D.L.T., Casanova R.d.J.P., Pifarre K.T., Ashraf I. (2022). An Effective Method for Lung Cancer Diagnosis from CT Scan Using Deep Learning-Based Support Vector Network. Cancers.

[21] Shafi I., Ansari S., Din S., Jeon G., Paul A. (2021). Artificial Neural Networks as Clinical Decision Support Systems. Proceedings of the Concurrency and Computation: Practice and Experience.

[22] Ramgopal S., Sanchez-Pinto L.N., Horvat C.M., Carroll M.S., Luo Y., Florin T.A. (2023). Artificial Intelligence-Based Clinical Decision Support in Pediatrics. Pediatr. Res..

[23] Ferdush J., Begum M., Hossain S.T. (2024). ChatGPT and Clinical Decision Support: Scope, Application, and Limitations. Ann. Biomed. Eng..

[24] Liao Z., Wang J., Shi Z., Lu L., Tabata H. (2024). Revolutionary Potential of ChatGPT in Constructing Intelligent Clinical Decision Support Systems. Ann. Biomed. Eng..

[25] Shafi I., Ansari S., Din S., Ashraf I. (2024). Cancer Detection and Classification Using a Simplified Binary State Vector Machine. Med. Biol. Eng. Comput..

[26] Casal-Guisande M., Fernández-Villar A., Mosteiro-Añón M., Comesaña-Campos A., Cerqueiro-Pequeño J., Torres-Durán M. (2024). Integrating Tabular Data through Image Conversion for Enhanced Diagnosis: A Novel Intelligent Decision Support System for Stratifying Obstructive Sleep Apnoea Patients Using Convolutional Neural Networks. Digit. Health.

[27] Côrte-Real J., Dutra I., Rocha R., Lachiche N., Vrain C. (2018). On Applying Probabilistic Logic Programming to Breast Cancer Data. Proceedings of the Inductive Logic Programming.

[28] Ferreira P., Dutra I., Salvini R., Burnside E. (2016). Interpretable Models to Predict Breast Cancer. Proceedings of the 2016 IEEE International Conference on Bioinformatics and Biomedicine, BIBM 2016.

[29] Casal-Guisande M., Comesaña-Campos A., Dutra I., Cerqueiro-Pequeño J., Bouza-Rodríguez J.-B. (2022). Design and Development of an Intelligent Clinical Decision Support System Applied to the Evaluation of Breast Cancer Risk. J. Pers. Med..

[30] Casal-Guisande M., Comesaña-Campos A., Núñez-Fernández M., Torres-Durán M., Fernández-Villar A. (2024). Proposal and Definition of an Intelligent Clinical Decision Support System Applied to the Prediction of Dyspnea after 12 Months of an Acute Episode of COVID-19. Biomedicines.

[31] Corbacho-Abelaira D., Casal-Guisande M., Corbacho-Abelaira F., Arnaiz-Fernandez M., Trinidad-Lopez C., Delgado Sanchez-Gracian C., Sanchez-Montanes M., Ruano-Ravina A., Fernandez-Villar A. (2024). Proposal and Definition of an Intelligent Decision- Support System Based on Deep Learning Techniques for the Management of Possible COVID-19 Cases in Patients Attending Emergency Departments. IEEE Access.

[32] Casal-Guisande M., Cerqueiro-Pequeño J., Comesaña-Campos A., Bouza-Rodríguez J.B. Proposal of a Methodology Based on Expert Systems for the Treatment of Diabetic Foot Condition. Proceedings of the Eighth International Conference on Technological Ecosystems for Enhancing Multiculturality, Association for Computing Machinery.

[33] Casal-Guisande M., Ceide-Sandoval L., Mosteiro-Añón M., Torres-Durán M., Cerqueiro-Pequeño J., Bouza-Rodríguez J.-B., Fernández-Villar A., Comesaña-Campos A. (2023). Design of an Intelligent Decision Support System Applied to the Diagnosis of Obstructive Sleep Apnea. Diagnostics.

[34] Casal-Guisande M., Torres-Durán M., Mosteiro-Añón M., Cerqueiro-Pequeño J., Bouza-Rodríguez J.-B., Fernández-Villar A., Comesaña-Campos A. (2023). Design and Conceptual Proposal of an Intelligent Clinical Decision Support System for the Diagnosis of Suspicious Obstructive Sleep Apnea Patients from Health Profile. Int. J. Environ. Res. Public Health.

[35] Casal-Guisande M., Comesaña-Campos A., Cerqueiro-Pequeño J., Bouza-Rodríguez J.B. (2022). Design and Definition of a New Decision Support System Aimed to the Hierarchization of Patients Candidate to Be Admitted to Intensive Care Units. Healthcare.

[36] Casal-Guisande M., Comesaña-Campos A., Cerqueiro-Pequeño J., Bouza-Rodríguez J.-B. (2020). Design and Development of a Methodology Based on Expert Systems, Applied to the Treatment of Pressure Ulcers. Diagnostics.

[37] Gayap H.T., Akhloufi M.A. (2024). Deep Machine Learning for Medical Diagnosis, Application to Lung Cancer Detection: A Review. BioMedInformatics.

[38] Zheng L., Eniola E., Wang J. Machine Learning for Colorectal Cancer Risk Prediction. Proceedings of the 2021 International Conference on Cyber-Physical Social Intelligence, ICCSI 2021.

[39] Ghazal S., Sauthier M., Brossier D., Bouachir W., Jouvet P.A., Noumeir R. (2019). Using Machine Learning Models to Predict Oxygen Saturation Following Ventilator Support Adjustment in Critically Ill Children: A Single Center Pilot Study. PLoS ONE.

[40] Kassania S.H., Kassanib P.H., Wesolowskic M.J., Schneidera K.A., Detersa R. (2021). Automatic Detection of Coronavirus Disease (COVID-19) in X-Ray and CT Images: A Machine Learning Based Approach. Biocybern. Biomed. Eng..

[41] Jiang Y., Yang Z.G., Wang J., Shi R., Han P.L., Qian W.L., Yan W.F., Li Y. (2022). Unsupervised Machine Learning Based on Clinical Factors for the Detection of Coronary Artery Atherosclerosis in Type 2 Diabetes Mellitus. Cardiovasc. Diabetol..

[42] Smith L.A., Oakden-Rayner L., Bird A., Zeng M., To M.S., Mukherjee S., Palmer L.J. (2023). Machine Learning and Deep Learning Predictive Models for Long-Term Prognosis in Patients with Chronic Obstructive Pulmonary Disease: A Systematic Review and Meta-Analysis. Lancet Digit. Health.

[43] Feng Y., Wang Y., Zeng C., Mao H. (2021). Artificial Intelligence and Machine Learning in Chronic Airway Diseases: Focus on Asthma and Chronic Obstructive Pulmonary Disease. Int. J. Med. Sci..

[44] Liao K.M., Liu C.F., Chen C.J., Shen Y.T. (2021). Machine Learning Approaches for Predicting Acute Respiratory Failure, Ventilator Dependence, and Mortality in Chronic Obstructive Pulmonary Disease. Diagnostics.

[45] Khanna V.V., Chadaga K., Sampathila N., Prabhu S., Rajagopala Chadaga P. (2023). A Machine Learning and Explainable Artificial Intelligence Triage-Prediction System for COVID-19. Decis. Anal. J..

[46] Goto T., Jo T., Matsui H., Fushimi K., Hayashi H., Yasunaga H. (2019). Machine Learning-Based Prediction Models for 30-Day Readmission after Hospitalization for Chronic Obstructive Pulmonary Disease. COPD J. Chronic Obstr. Pulm. Dis..

[47] Liew C.Q., Chen Y.P., Gao J.W., Ko C.H., Tsai C.L. (2024). Predicting COPD Readmissions: A Novel 2e Index with Traditional Regression and Machine-Learning Approaches. Intern. Emerg. Med..

[48] Wang L., Li G., Ezeana C.F., Ogunti R., Puppala M., He T., Yu X., Wong S.S.Y., Yin Z., Roberts A.W. (2022). An AI-Driven Clinical Care Pathway to Reduce 30-Day Readmission for Chronic Obstructive Pulmonary Disease (COPD) Patients. Sci. Rep..

[49] Li M., Cheng K., Ku K., Li J., Hu H., Ung C.O.L. (2023). Modelling 30-Day Hospital Readmission after Discharge for COPD Patients Based on Electronic Health Records. NPJ Prim. Care Respir. Med..

[50] Zhang R., Lu H., Chang Y., Zhang X., Zhao J., Li X. (2022). Prediction of 30-Day Risk of Acute Exacerbation of Readmission in Elderly Patients with COPD Based on Support Vector Machine Model. BMC Pulm. Med..

[51] Lopez K., Li H., Lipkin-Moore Z., Kay S., Rajeevan H., Davis J.L., Wilson F.P., Rochester C.L., Gomez J.L. (2023). Deep Learning Prediction of Hospital Readmissions for Asthma and COPD. Respir. Res..

[52] Verma V.K., Lin W.Y. (2022). Machine Learning-Based 30-Day Hospital Readmission Predictions for COPD Patients Using Physical Activity Data of Daily Living with Accelerometer-Based Device. Biosensors.

[53] Mohamed I., Fouda M.M., Hosny K.M. (2022). Machine Learning Algorithms for COPD Patients Readmission Prediction: A Data Analytics Approach. IEEE Access.

[54] Fernández Villar A., Golpe Gómez R., González Montaos A., Fernández García S., Pazos Area L., Priegue Carrera A., Ruano Raviña A., Represas Represas C. (2023). The Impact of the SARS-CoV-2 Pandemic on the Demographic, Clinical and Social Profiles of Patients Admitted to the Pneumology Department for a COPD Exacerbation. PLoS ONE.

[55] Fernández-García S., Represas-Represas C., Ruano-Raviña A., Mouronte-Roibás C., Botana-Rial M., Ramos-Hernández C., Fernández-Villar A. (2020). Social and Clinical Predictors of Short- and Long-Term Readmission after a Severe Exacerbation of Copd. PLoS ONE.

[56] Galli S. (2022). Feature Selection in Machine Learning with Python.

[57] Jeon H., Oh S. (2020). Hybrid-Recursive Feature Elimination for Efficient Feature Selection. Appl. Sci..

[58] Flach P.A. (2013). Machine Learning: The Art and Science of Algorithms That Make Sense of Data.

[59] Mamdani E.H., Assilian S. (1975). An Experiment in Linguistic Synthesis with a Fuzzy Logic Controller. Int. J. Man Mach. Stud..

[60] Mamdani E.H. (1976). Advances in the Linguistic Synthesis of Fuzzy Controllers. Int. J. Man Mach. Stud..

[61] Mamdani E.H. (1977). Application of Fuzzy Logic to Approximate Reasoning Using Linguistic Synthesis. IEEE Trans. Comput..

[62] Ross T.J. (2010). Fuzzy Logic with Engineering Applications.

[63] Wang L.X., Mendel J.M. (1992). Generating Fuzzy Rules by Learning from Examples. IEEE Trans. Syst. Man Cybern..

[64] Casal-Guisande M., Cerqueiro-Pequeño J., Bouza-Rodríguez J.-B., Comesaña-Campos A. (2023). Integration of the Wang & Mendel Algorithm into the Application of Fuzzy Expert Systems to Intelligent Clinical Decision Support Systems. Mathematics.

[65] Boughorbel S., Jarray F., El-Anbari M. (2017). Optimal Classifier for Imbalanced Data Using Matthews Correlation Coefficient Metric. PLoS ONE.

[66] Chicco D., Jurman G. (2020). The Advantages of the Matthews Correlation Coefficient (MCC) over F1 Score and Accuracy in Binary Classification Evaluation. BMC Genom..

[67] Guilford J.P. (1954). Psychometric Methods.

